# A newly isolated human intestinal bacterium strain capable of deglycosylating flavone *C*-glycosides and its functional properties

**DOI:** 10.1186/s12934-019-1144-7

**Published:** 2019-05-28

**Authors:** Shiqi Zheng, Di Geng, Shuangyue Liu, Qingqing Wang, Siqi Liu, Rufeng Wang

**Affiliations:** School of Life Sciences, Beijing University of Chinese Medicine, Beijing, 102488 China

**Keywords:** *Enterococcus faecalis*, Flavone *C*-glycosides, Deglycosylation, Reduced carbon nutrition source induction

## Abstract

**Background:**

Flavone *C*-glycosides are difficult to be deglycosylated using traditional chemical methods due to their solid carbon–carbon bond between sugar moieties and aglycones; however, some bacteria may easily cleave this bond because they generate various specific enzymes.

**Results:**

A bacterial strain, named W12-1, capable of deglycosylating orientin, vitexin, and isovitexin to their aglycones, was isolated from human intestinal bacteria in this study and identified as *Enterococcus faecalis* based on morphological examination, physiological and biochemical identification, and 16S rDNA sequencing. The strain was shown to preferentially deglycosylate the flavone *C*-glycosides on condition that the culture medium was short of carbon nutrition sources such as glucose and starch, and its deglycosylation efficiency was negatively correlated with the content of the latter two substances.

**Conclusion:**

This study provided a new bacterial resource for the cleavage of *C*-glycosidic bond of flavone *C*-glycosides and reported the carbon nutrition sources reduction induced deglycosylation for the first time.

**Electronic supplementary material:**

The online version of this article (10.1186/s12934-019-1144-7) contains supplementary material, which is available to authorized users.

## Background

Flavone *C*-glycosides refer to a group of compounds of which the sugar moieties are usually connected to C-6 or/and C-8 of the flavone skeletons through a C–C bond [1, 2]. Together with the *C*-glycosides containing other flavonoid skeletons such as isoflavone, chalcone and flavonol, they are also called flavonoid *C*-glycosides. Most of the flavone *C*-glycosides hitherto found have luteolin or apigenin as aglycones, and their sugar moieties frequently include glucosyl, galactosyl, arabinosyl, rhamnosyl, apiosyl, xylosyl, and rutinosyl groups [3]. Orientin, vitexin, isoorentin, isovitexin and their derivatives are considered as the most common flavone *C*-glycosides in nature [3, 4]. Flavone *C*-glycosides are mainly distributed in plants and constitute the important components in human diets like cereals, fruits, and so on [4]. These compounds benefit the human health because they display diverse biological activities such as antioxidant, anti-inflammatory, antiviral, antibacterial, hepatoprotective, antidepressant and antispasmodic effects [1, 3, 5–10]. The bioactivities of flavone *C*-glycosides are usually attributed to their aglycones because the latter are absorbed faster than the glycoside forms [11]. Thus, the glycoside forms generally have a low bioavailability especially when they are used orally [11–14]. It has been known that deglycosylation is regarded as a crucial process for improving absorption of glycosides in vivo. After deglycosylation, the resulted aglycones can be absorbed into blood circulation through epithelium of small intestine [15, 16]. The flavone *O*-glycosides are usually deglycosylated much easily due to the unstable *O*-glycosidic bond. In contrast, the flavone *C*-glycosides appear very stable and difficult to be cleaved in normal condition because of the stability of *C*-glycosidic bond [17, 18].

Although it is hard to deglycosylate the flavone *C*-glycosides in regular environment, this can be done sometimes in human alimentary tract [19, 20]. It has been well known that the human intestinal tract, in particular colon accommodates a complex ecosystem including numerous gut bacteria which play a significant role in maintaining host health status [21, 22]. Glycosides inevitably interact with the bacteria in the intestinal tract in the case of oral administration. Some species of these microbes are capable of mediating the cleavage of glycosides to their aglycones by means of deglycosylating enzymes. This reaction is considered to improve the bioavailability and bioactivities of glycosides [21, 23–25]; meanwhile, the resultant sugar molecules can provide carbon nutrition source for the intestinal flora [26]. Even so, the intestinal bacteria which are able to deglycosylate flavone *C*-glycosides have been found rarely and the metabolism of *C*-glycosides mediated by these bacteria has been studied to a lesser extent [11, 19, 20, 27]. Therefore, it is informative and meaningful to find these unusual microbes and study the characteristics of the reaction catalyzed by them.

## Results

### Screen and isolation of target strain

**Fig. 1:**
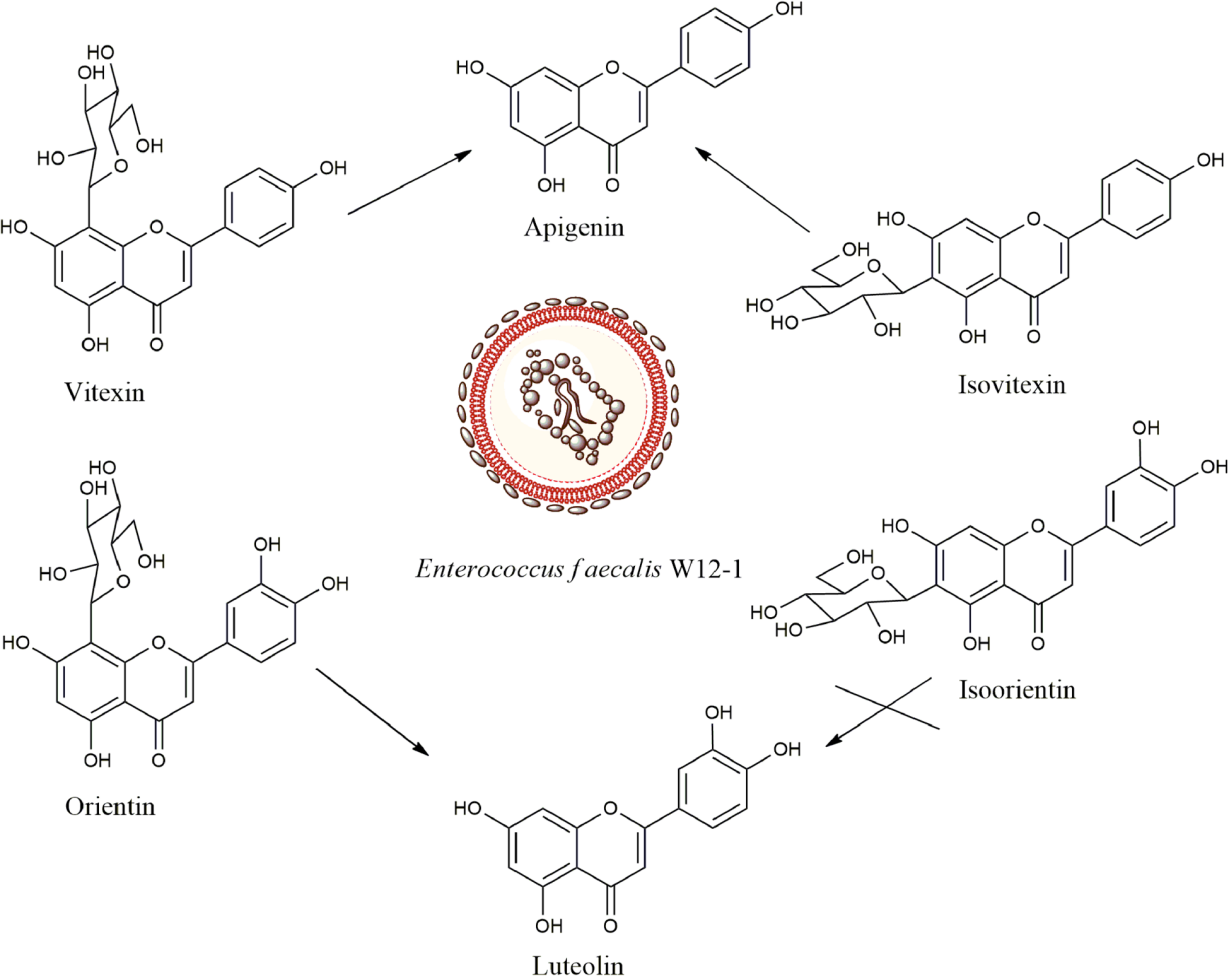
Structures and transformation of the compounds of interest

**Fig. 2:**
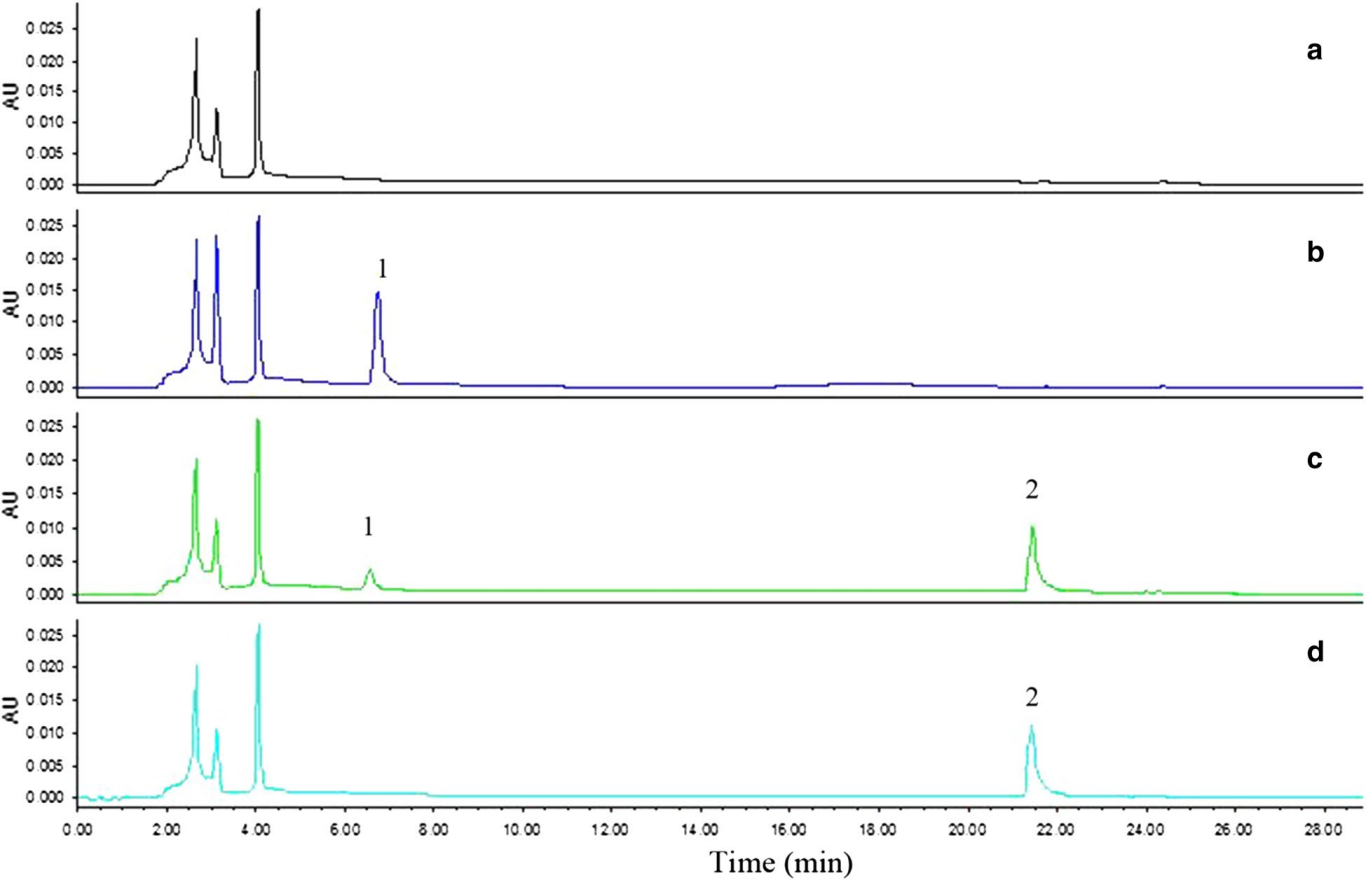
HPLC chromatograms related to deglycosylation of orientin. **a** blank group; **b** experimental group at 0 h; **c** reference compounds of orientin and luteolin in GAM; **d** experimental group at 24 h. 1: orientin; 2: luteolin

The fecal samples from 10 healthy young people were screened by deglycosylation test in combination with activity assay. As a result, only one sample from a 25-year-old male volunteer was found to deglycosylate orientin and vitexin (Fig. 1). This sample was then isolated by plate streaking, and about 50 bacterial colonies which had developed on the plates were picked up. Among them, a colony which could transform orientin to luteolin, the aglycone of the former, was further purified by repeated plate streaking to obtain a strain W12-1. Like its parent colony, this strain could also deglycosylate orientin. The products of deglycosylation were detected by high performance liquid chromatography (HPLC), and the retention times of orientin and luteolin were 6.5 and 21.4 min, respectively (Fig. 2).

### Identification of isolate

#### Morphological examination and physiological and biochemical identification

**Fig. 3:**
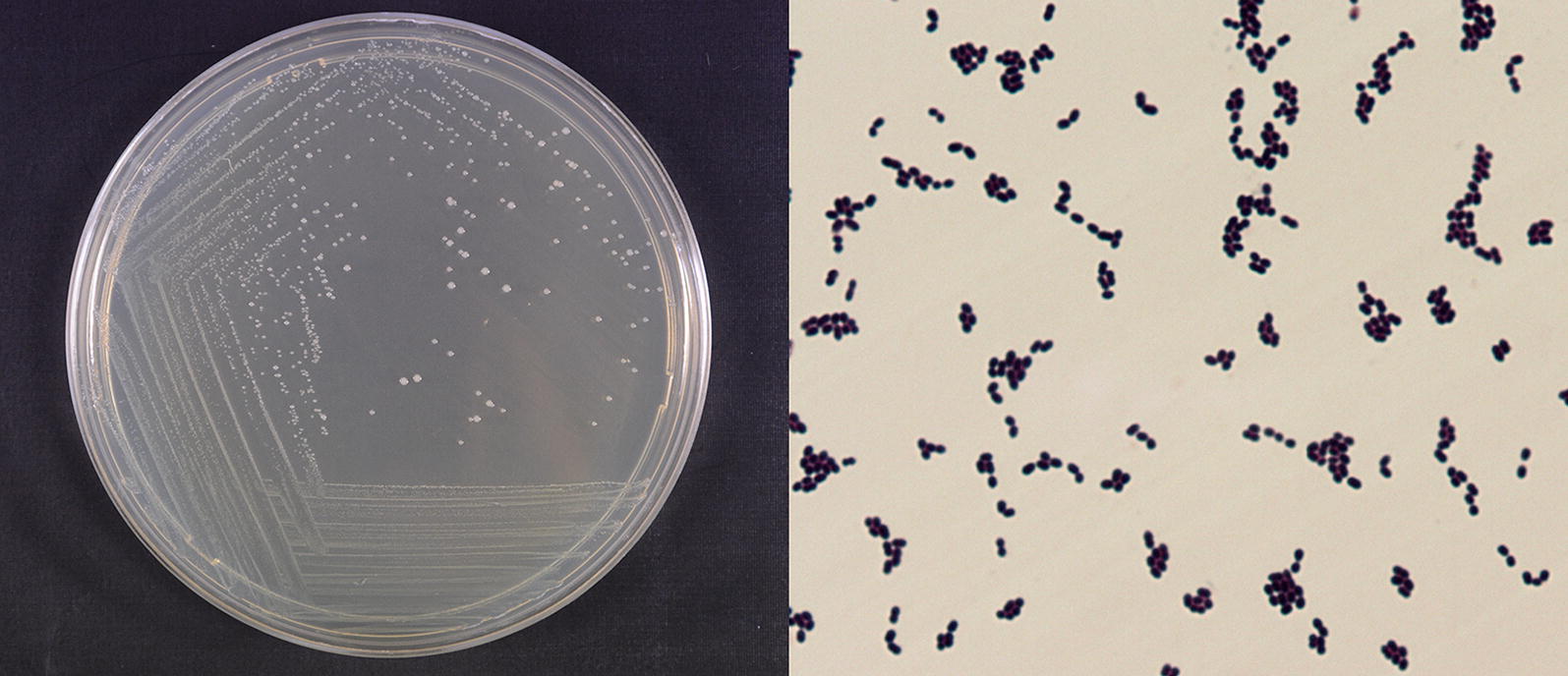
Morphological characteristics of strain W12-1. Left: the colony; Right: the cells

Strain W12-1 was a facultative anaerobic Gram-positive strain which was evidenced by Gram staining. Morphological observation indicated that its colony on trypticase-peptone-yeast extract (TPY) agar plate was creamy-white, translucent, and humid on the surface, and the individual bacterial cells were ellipsoidal and about 0.5–1.0 μm in diameter (Fig. 3). The detailed physiological and biochemical properties of the strain are shown in Additional file 1: Table S1.

#### 16S rDNA sequencing

**Fig. 4:**
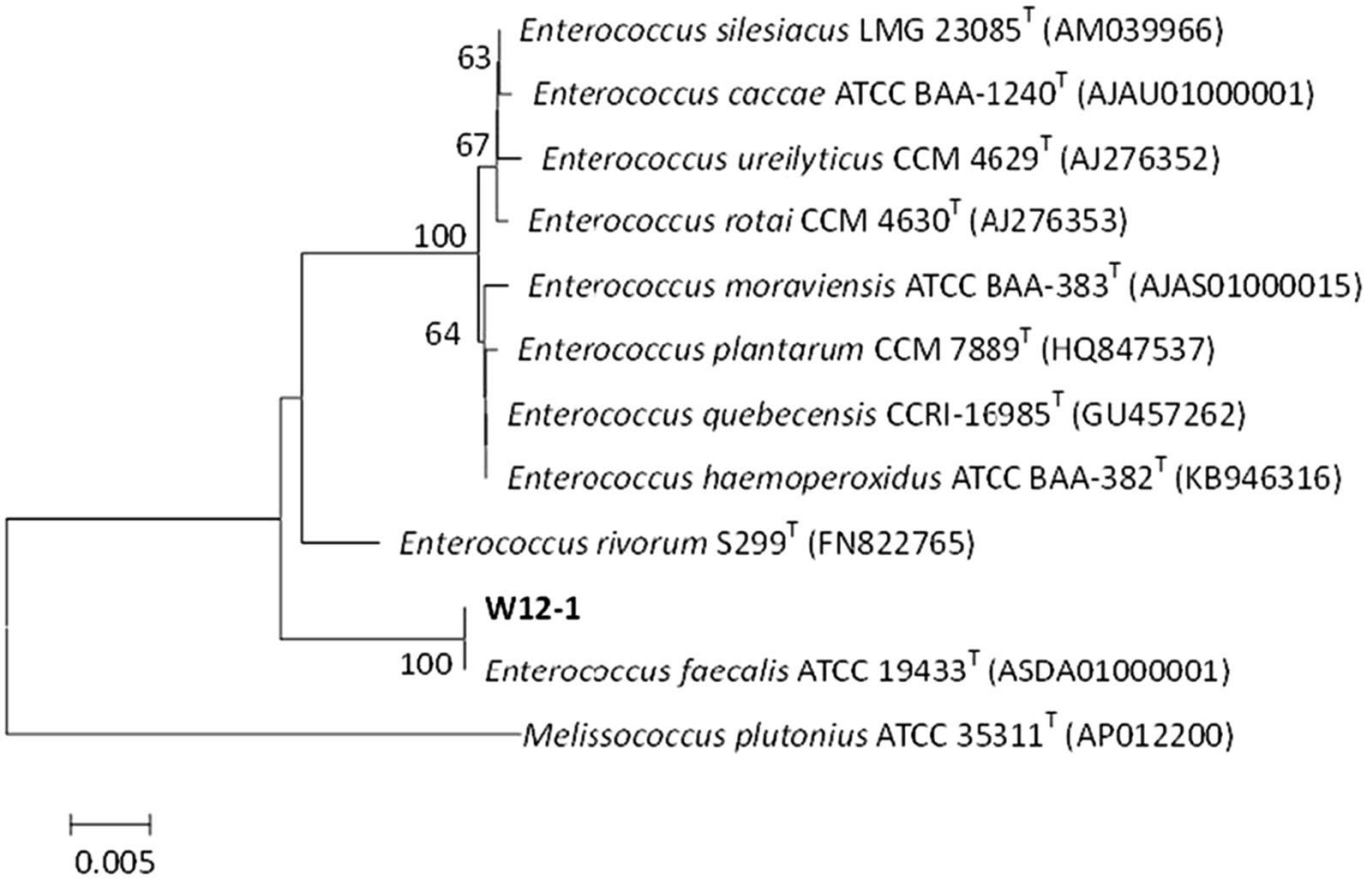
Neighbor-joining tree based on 16S rDNA gene sequences

The genomic DNA of strain W12-1 was obtained and the 16S rDNA gene sequence with 1398 base pairs was almost completely amplified with primers using polymerase chain reaction (PCR). The resultant 16S rDNA nucleotide sequence has been deposited in the GenBank under accession No. J67GGCKP01R. After comparing the 16S rDNA gene sequence of this strain with that in the databank using CLUSTAL W program in accordance with the neighbor-joining method [28, 29], the phylogenetic affiliation of the bacterium was determined (Fig. 4). It showed 97–100% sequence similarity to all *Enterococcus* species examined and had 100% sequence similarity to *E. faecalis* ATCC 19433 (100%, ASDA01000001). Based on the phylogenetic tree, the isolate was placed in the same cluster as *E. faecalis* and its identity was determined.

#### Deglycosylation efficiency

The biotransformation results showed that strain W12-1 deglycosylated orientin to luteolin, and deglycosylated both vitexin and isovitexin to apigenin; however, it did not transform isoorientin and puerarin to their corresponding aglycones (Fig. 1).

**Fig. 5:**
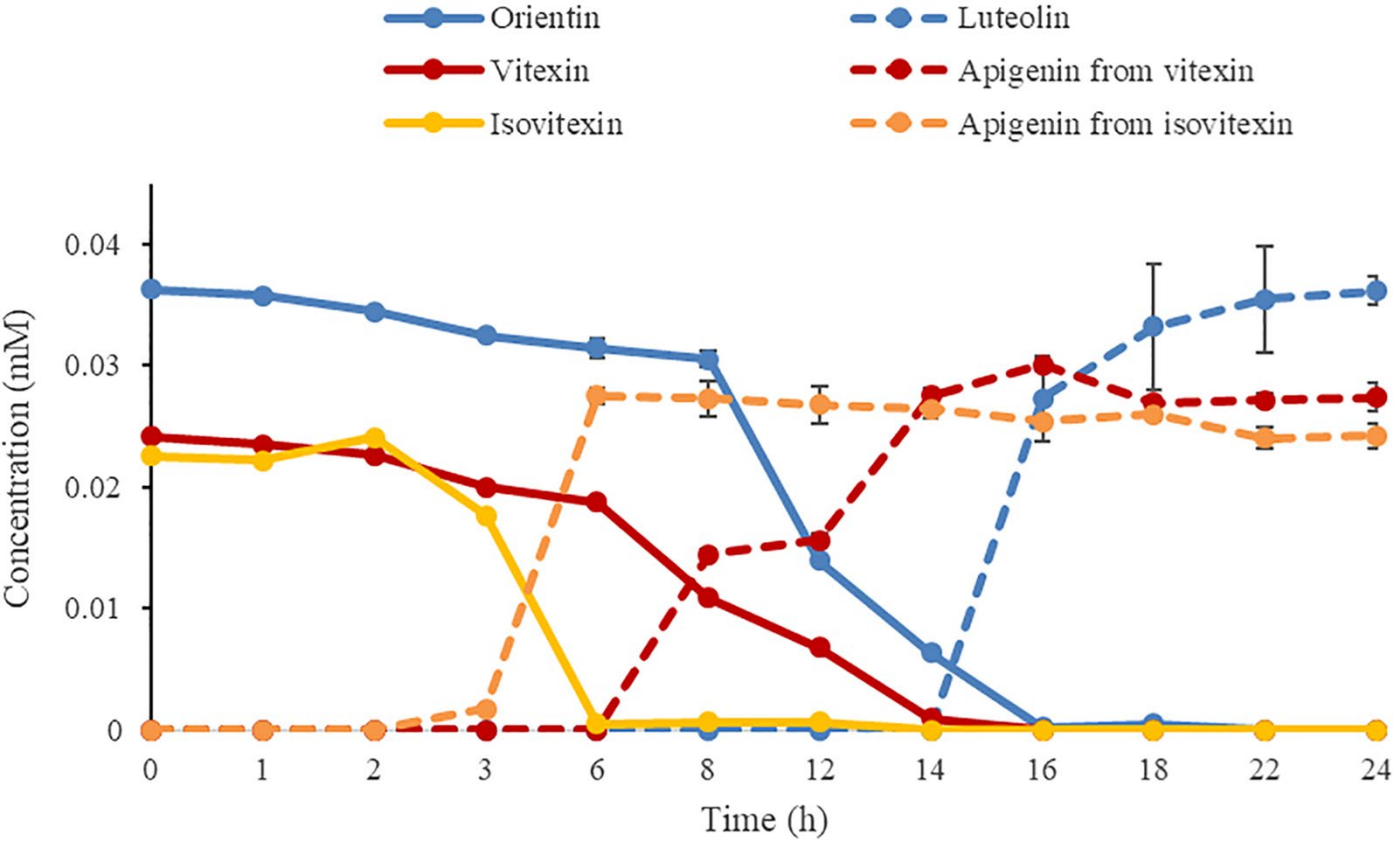
Time course of deglycosylation of orientin, vitexin and isovitexin (n = 3, mean ± SD)

The time-dependent curve (Fig. 5) showed that the efficiencies of strain W12-1 to deglycosylate orientin, vitexin and isovitexin were different. The deglycosylation of isovitexin was faster than that of orientin and vitexin in the same case. The strain transformed isovitexin to apigenin completely within 6 h, whereas it transformed orientin and vitexin to their aglycones within about 16 and 14 h, respectively. In any case, all of these substrates could be deglycosylated eventually by this strain in 24 h.

#### Influence of carbon nutrition source on deglycosylation

**Fig. 6:**
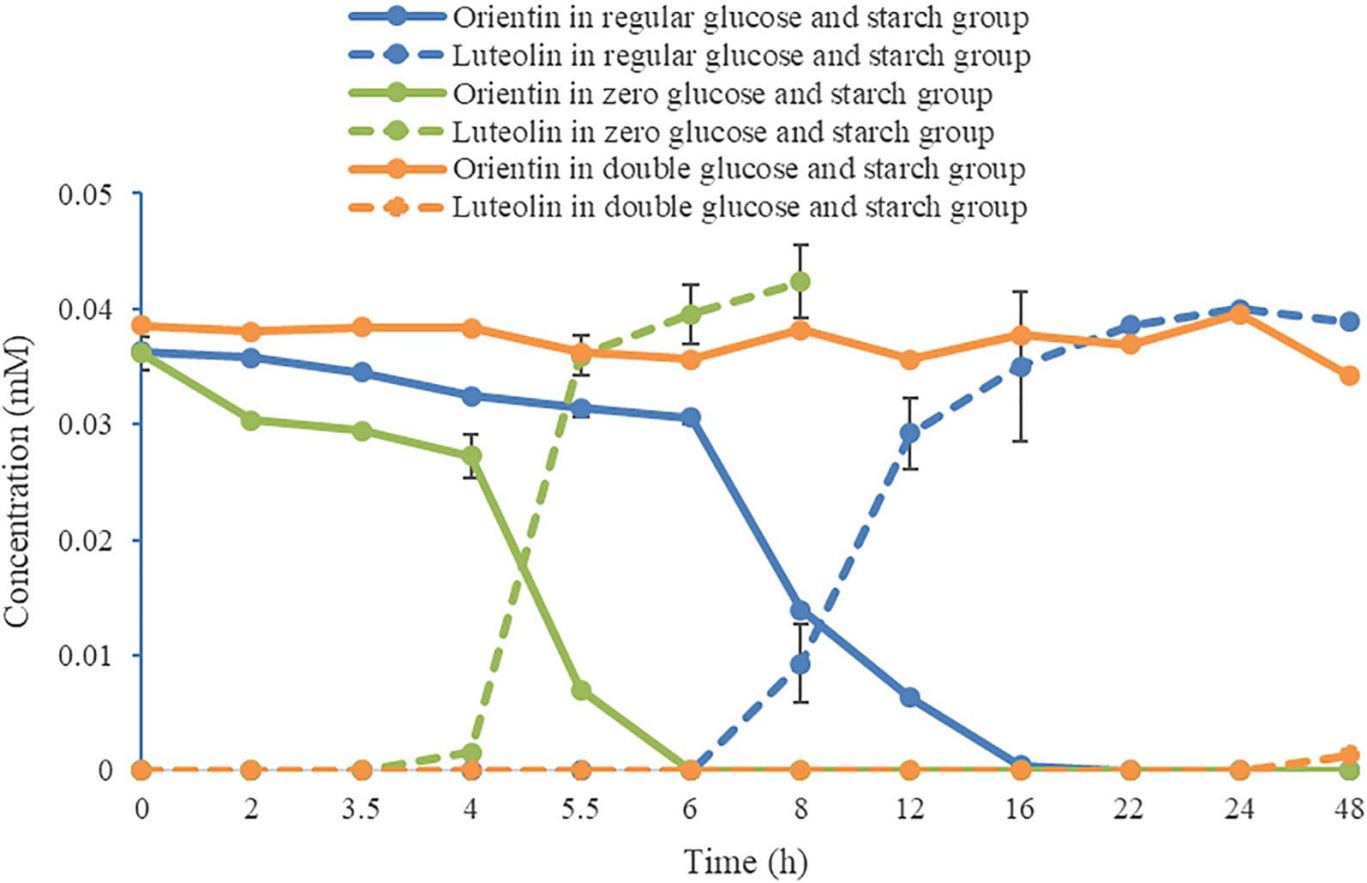
Time course of deglycosylation of orientin in the media with different contents of carbon nutrition (n = 3, mean ± SD)

As shown in Fig. 6, the content of carbon nutrition sources including glucose and soluble starch has a crucial influence on the deglycosylation efficiency of the strain. The strain quickly transformed orientin to luteolin within about 6 h in the absence of glucose and starch in the medium. Nevertheless, the deglycosylation efficiency of the strain was significantly lower in the medium with regular level of glucose and soluble starch. Moreover, the strain almost could not deglycosylate orientin in 48 h as the level of glucose and soluble starch was doubled (Additional file 2: Table S2).

#### Influence of carbon nutrition source on enzyme profiling

**Fig. 7:**
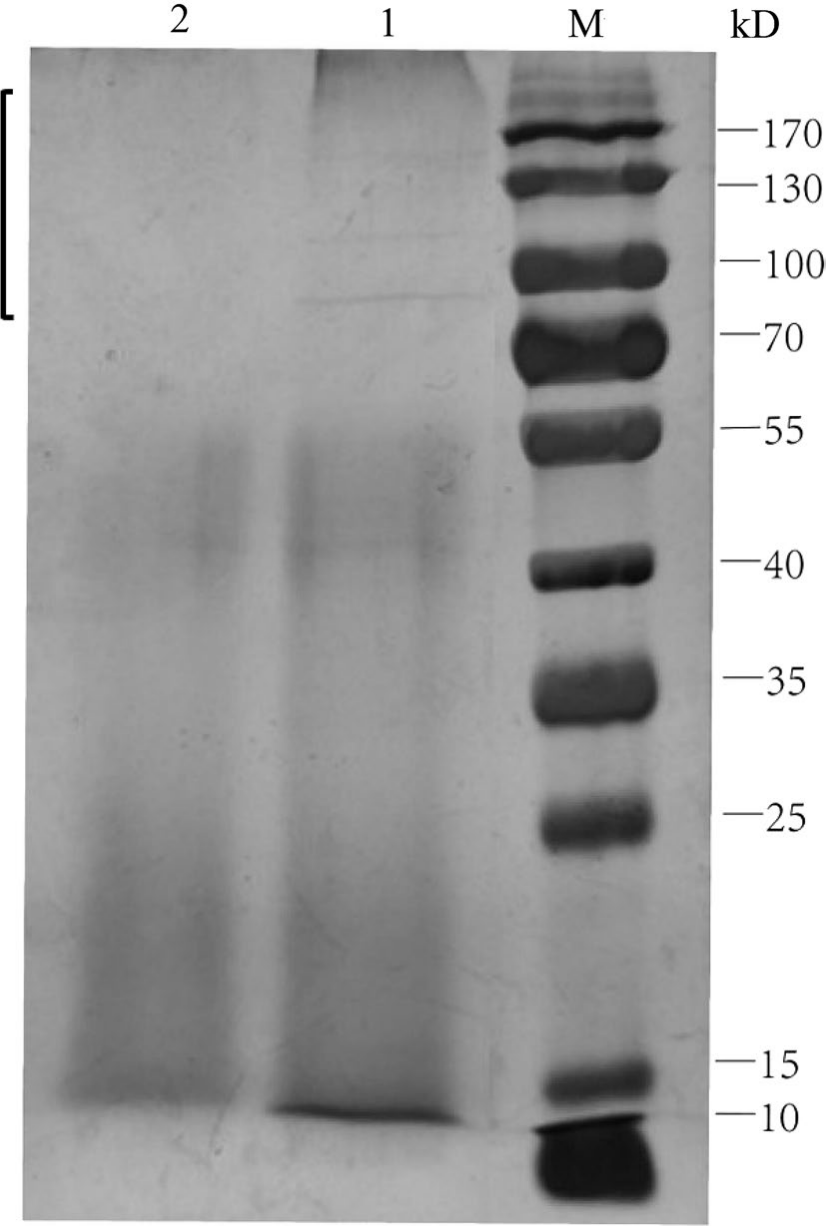
Comparative SDS-PAGE electrophoretogram of strain W 12-1 culture in the media with double content of carbon sources and without carbon sources. M: protein ladder; 1: the culture in the medium without carbon sources; 2: the culture in the medium with double content level of carbon sources

The electrophoretogram (Fig. 7) showed that the enzyme profile of strain W12-1 cultured in the medium without carbon nutrition source differs significantly from that of the strain cultured in the medium with double level of carbon nutrition source in the region above 70 kDa. Under the same concentration, the protein bands in this area in the samples without carbon nutrition source were significantly clear, while those in the samples with double level of carbon nutrition source were unclear to even absent.

## Discussion

Glycosides are considered as one of the main carbon nutrition sources for intestinal flora. They are cleaved to the corresponding aglycones in the intestinal tract, and the free sugar groups are utilized by the intestinal flora. The enzymes produced by intestinal bacteria provide unique advantage for their utilization of glycoside compounds, which is also a characteristic of intestinal floral metabolism. However, the intestinal bacteria which are able to cleave flavone *C*-glycosides have been reported infrequently, and their deglycosylating properties were hardly known. In this study, we obtained a mixed human intestinal flora through massive screening and isolated a pure strain W12-1 which could deglycosylate some flavone *C*-glycosides to their aglycones. According to the results of the morphological examination, physiological and biochemical identification and 16s rDNA sequencing, the isolate was identified as *E. faecalis* (Fig. 4). This is the first time that the species of *Enterococcus* has been found to be able to deglycosylate flavone *C*-glycosides. In the same genus, only *E. faecium* and *E. sp.* MRG-IFC-2 were reported to deglycosylate isoflavone *C*-glycoside puerarin [30, 31]. Most of the previously reported active species capable of deglycosylating flavonoid *C*-glycosides were from *Eubacterium*, *Bacteroides*, *Lactococcus*, and some genera of Lachnospiraceae, for example, *Clostridium* [26, 32–34].

This strain exhibited somewhat specific deglycosylation activity. Among the compounds tested, it could deglycosylate flavone 8-*C*-glycosides such as orientin and vitexin, but selectively deglycosylate flavone 6-*C*-glycosides. Interestingly, although this strain could not deglycosylate isoorientin, it could deglycosylate isovitexin faster than deglycosylate orientin and vitexin (Figs. 1, 5).

We found that the content of carbon sources in the media was negatively correlated with the deglycosylation efficiency of the strain under the same concentration. The strain cultured in the medium with double content level of glucose and starch had almost no deglycosylation activity in 24 h, whereas that cultured in the media with regular content level and zero content level of glucose and starch totally deglycosylated the *C*-glycosides in 6–20 h. Furthermore, the less the glucose and starch were contained in the media, the faster the deglycosylation was performed. This is the first report that the deduction of carbon sources can induce and enhance the capacity of bacteria to deglycosylate *C*-glycosides. It was also found that the initiation of deglycosylation needs an activation period of which the duration also depends on the quantity of carbon sources (Fig. 6). The differential capacity and initiation of the strain to deglycosylate *C*-glycosides reflected that it prefers utilizing free carbon sources in culture medium to cleaving the sugar from substrates. This may be an adaptation or makeshift of the strain under harsh conditions such as shortage of carbon sources, e.g. glucose and starch. The electrophoretogram demonstrated that more proteins were produced when the glucose and starch were reduced (Fig. 7). It is suggested that the quantity of enzymes responsible for the cleavage of flavone *C*-glycosides produced by the strain was increased under harsh conditions, and the control of the carbon sources is a practical way to regulate the production of these enzymes.

## Conclusion

We first reported that a strain of *E. faecalis* can deglycosylate orientin, vitexin, and isovitexin, and its deglycosylating capacity is negatively correlated with the content of carbon sources, e.g. glucose and starch in the medium. The results laid a foundation for further investigating the enzymes that could efficiently cleave flavone *C*-glycosides.

## Materials and methods

### Apparatus

An YQX-II anaerobic incubator manufactured by Shanghai Cimomedical Appliance Company (Shanghai, China) and a CY-12C digital oxygen meter which was the product of Zhejiang Xinanjiang No. 2 Analytical Instrument Factory (Hangzhou, China) were used to cultivate the intestinal bacteria and monitor the anaerobic environment. The bacterial cultures were centrifuged by a TGL-20 M low temperature and high speed centrifuge made by Shanghai Luxiangyi Centrifuge Instrument Co., Ltd. (Shanghai, China). HPLC was performed on a Waters e2695 series instrument manufactured by Waters Inc. (Milford, USA) equipped with a 2489 UV/Visible detector and a conditioned autosampler at 4 °C. The 16S rDNA was amplified using a GeneAmp^®^ PCR System 9700 manufactured by Thermo Fisher Scientific (Waltham, USA). Electrophoresis was conducted on a Mini-PROTEAN^®^ Tetra vertical electrophoresis cell which was the product of Bio-Rad (Hercules, USA).

### Reagents and chemicals

The HPLC-grade acetonitrile obtained from Fisher Co. (Pittsburgh, USA), and Wahaha ultrapure water manufactured by Hangzhou Wahaha Group Co., Ltd. (Hangzhou, China) were employed to prepare mobile phases. The biochemical reagents for general anaerobic medium (GAM) preparation including tryptone, soya peptone, proteose peptone, digestive serum powder, yeast extract, beef extract, beef liver extract powder, l-cysteine hydrochloride, soluble starch etc. were purchased from Beijing Aoboxing Biotechnique Co., Ltd. (Beijing, China). The bacterial genomic DNA extraction kit was provided by Beijing Dingguo Changsheng Biotech Co., Ltd. (Beijing, China) and the DNA gel extraction kit was manufactured by Beijing Biomed Co., Ltd. (Beijing, China). The PAGE gel silver staining kit (Cat. No. G7210) was supplied by Beijing Solarbio Science & Technology Co., Ltd. (Beijing, China) and the protein ladder (Cat. No. 26616) was the product of Thermo Scientific (Shanghai, China). The reference compounds with the purities above 99% used in the experiments including orientin, vitexin, isoorientin, isovitexin, luteolin, apigenin and puerarin were supplied by National Institutes for Food and Drug Control (Beijing, China). Other chemicals such as acetic acid, glucose, sodium thioglycolate, dipotassium hydrogen phosphate, sodium chloride, sodium hydroxide, etc. were the products of Beijing Chemical Works (Beijing, China).

### Isolation of human intestinal bacteria

#### Preparation of general anaerobic medium

The GAM was prepared in two steps. Firstly, solutions A, B and C were separately prepared. For solution A, 10.0 g of tryptone, 13.5 g of digestive serum powder, 10.0 g of proteose peptone, 3.0 g of soya peptone, 1.2 g of beef liver extract powder, 5.0 g of yeast extract, 2.2 g of beef extract, and appropriate volume of distilled water were placed in a 500 mL flask and stirred under heating until the substances were completely dissolved. For solution B, 3.0 g of glucose, 5.0 g of soluble starch, 2.5 g of dipotassium hydrogen phosphate, 3.0 g of sodium chloride, and appropriate volume of distilled water were put into a 250 mL flask and stirred under heating until the substances were completely dissolved. For solution C, 0.3 g of l-cysteine hydrochloride, 0.3 g of sodium thioglycolate, and a quantity of distilled water were added into a 100 mL flask and stirred until the substances were completely dissolved. Secondly, above three solutions were mixed together and made up to 1000 mL with distilled water. The resultant solution was adjusted to pH 7.3 using aqueous solution of sodium hydroxide; then, it was autoclaved at 121 °C for 20 min.

#### Preparation of intestinal flora

The collection and preparation of intestinal bacteria were performed in accordance with the reported standard operation procedures (SOP) [35]. The fresh human fecal samples were donated by 10 healthy volunteers including 5 males and 5 females aged from 22 to 25 years. These volunteers had not taken any medicine in 3 months and avoided using alcohol and food rich in polyphenols in 48 h before fecal collection. The fresh feces of the volunteers was collected and quickly put into a ziplock bag filled with nitrogen gas, which was extruded by hands to homogenize the feces. In the sterile anaerobic incubator, about 5 g of the feces was added into 200 mL of sterilized GAM medium and cultured at 37 °C for 24 h. Then, 1 mL of the bacterial suspension was removed and inoculated into 9 mL of fresh GAM medium, incubated again at 37 °C for 24 h in anaerobic condition to obtain the intestinal bacterial flora.

#### Screen of active intestinal flora

Before screening, 50 μL of the suspension of intestinal bacterial flora was added into 5 mL of fresh GAM medium and anaerobically cultured at 37 °C for 24 h to obtain the activated intestinal bacterial flora. Then, 120 µL of the suspension of activated intestinal bacterial flora was taken and incubated at 37 °C with 3 mL of fresh GAM medium containing the substrate compounds. The transformation activity was detected through activity assay.

#### Isolation of strain

The isolation of pure bacterial strains was conducted referring to a literature [36]. The activated bacterial mixture from an active sample was diluted 10-fold with normal saline and streaked onto GAM agar plates using an inoculating loop. The plates were sealed and placed upside down, and incubated anaerobically at 37 °C for 48 h. Then, the colonies developing on the plates were picked up on the basis of their shape and size and screened through activity assay. The active strains screened out were inoculated into the liquid GAM medium again, and the above procedures were repeated until the pure single strain was obtained.

### Activity assay

#### Preparation of test solutions of substrates

A quantity of orientin, vitexin and isovitexin were accurately weighed and dissolved in appropriate volume of dimethyl sulphoxide (DMSO) to obtain the stock solutions of each compound at 4.67, 4.75 and 5.08 mg/mL, respectively. Then, these stock solutions were diluted to the desired concentrations (0.1 mM) with GAM to obtain the respective test solutions.

#### Screen of active strain

The deglycosylation activity of the isolated single bacterial strains was primarily screened by incubating them with the test solution of orientin, and secondly screened by incubating them with the test solutions of vitexin and isovitexin, respectively. During the primary screen, a quantity of each bacterial strain was added into 3 mL of GAM broth containing 0.1 mM orientin, and the broth was incubated anaerobically at 37 °C for 48 h. After that, about 500 μL of the reaction mixture was centrifuged at 14,800 rpm and 4 °C for 15 min, and about 300 μL of the supernatant was collected and mixed with 900 μL of methanol. The mixture was vortexed and then further centrifuged at 14,800 rpm and 4 °C for 15 min in order to remove the proteins. The supernatant of the solution was collected and filtered through a 0.22 μm membrane filter, and the filtrate was analyzed by HPLC. The compound-free GAM solution was used as blank group, and the GAM solution containing the compound without bacterial strain was used as control group. All experiments were carried out in triplicate. During the second screen, the isolate capable of transforming orientin to luteolin was added into 3 mL of GAM broth containing 0.1 mM vitexin, isoorientin and isovitexin, respectively. The mixtures were incubated anaerobically at 37 °C for 48 h. The compound-free GAM solution was used as blank group, and the GAM solution containing the compound without bacterial strain was used as control group. All experiments were carried out in triplicate, and the sample processing was the same as that for primary screen.

#### HPLC analysis

The HPLC analysis was carried out on a Waters instrument with a Phenomenex C_18_ column (4 μm, 250 mm × 4.6 mm), and the temperature of column oven was maintained at 35 °C. The gradient elution including 20% A at 0–10 min, 20–40% A at 10–20 min, 40–45% A at 20–25 min, and 45–20% A at 25–29 min was conducted using acetonitrile as solvent A and 1% acetic acid in purified water as solvent B at a flow rate of 1 mL/min. The detection wavelength was 365 nm, and the injection volume of each run was 10 μL. The content of both glycosides and aglycones in various samples were calculated on the basis of the calibration curve for each compound (Table 1).

**Table 1:** Calibration curves of the analytes

Analytes	Retention time (min)	Calibration curve	Correlation coefficient (r)	Linear range (μg/mL)
Orientin	6.45	y = 2 × 10^7^x + 5591.1	0.9995	0.7–11.7
Vitexin	8.14	y = 2 × 10^7^x + 6476.1	0.9996	0.7–11.9
Isovitexin	8.59	y = 2 × 10^7^x + 6332.2	0.9995	0.8–12.7
Luteolin	21.42	y = 4 × 10^6^x − 696.9	0.9998	0.3–9.9
Apigenin	24.08	y = 3 × 10^6^x − 3777.3	0.9996	0.3–10.1

#### Identification of active isolate

An isolate W12-1 which showed deglycosylation activity in both primary screen and second screen was incubated on TPY agar plate and TPY solution in an anaerobic incubator at 37 °C for 24 h to carry out the morphological examination of the strain. Both physiological and biochemical characteristics of the strain were determined by China Center of Industrial Culture Collection (CICC, Beijing, China), which is a qualified authoritative facility to provide this service in China, in accordance with its own SOP. This isolate has been deposited at China General Microbiological Culture Collection Center (CGMCC) under deposition number of CGMCC 17244.

Genomic DNA was extracted from this isolate employing a bacterial genomic DNA extraction kit according to the instruction in its packaging insert. The 16S rDNA sequence of the strain was obtained by PCR using 2 universal primers, namely 27 F (5′-AGAGTTTGATCCTGGTGGCTCAG-3′) and 1492 R (5′-ACGGTTACCTTGTTACGACTT-3′), which were designed and synthesized by Sangon Biotech (Shanghai) Co., Ltd. (Shanghai, China). The PCR amplification program was as follows: initial denaturation at 95 °C for 2 min, followed by 35 cycles comprising denaturation at 95 °C for 1 min, annealing at 51 °C for 15 s, and extension at 72 °C for 1 min, and an additional extension step at 72 °C for 5 min. The PCR product was purified by agarose gel electrophoresis using a DNA gel extraction kit. Sequencing of the 16S rDNA fragments was conducted by Majorbio BioTech Co., Ltd. (Shanghai, China). A phylogenetic tree was constructed using CLUSTAL W program and MEGA (ver 5.0) software in accordance with the neighbor-joining method.

#### Deglycosylation efficiency test

The efficiencies of strain W12-1 to deglycosylate orientin, vitexin and isovitexin were comparatively investigated using a biotransformation system containing GAM, the strain, and the respective compounds at the concentrations of 0.1 mM. Each mixture was incubated anaerobically at 37 °C and stopped at 0, 1, 2, 3, 6, 8, 12, 14, 16, 18, 22 and 24 h, respectively. The blank group and control group were also set as those for activity assay. All experiments were carried out in triplicate, and the sample processing was the same as that for activity assay.

#### Test of carbon source deduction

The efficiency of strain W12-1 to deglycosylate orientin was also investigated in the GAM media with double (including 6.0 g of glucose and 10.0 g of soluble starch), regular (including 3.0 g of glucose and 5.0 g of soluble starch) and zero (without glucose and soluble starch) content of carbon sources, respectively. The experiments were performed using a similar biotransformation system as above except the content of carbon sources, and sampled at 0, 2, 3.5, 4, 5.5, 6, 8, 12, 16, 22, 24 and 48 h, respectively.

#### SDS-PAGE

The cell cultures in GAM media containing double carbon sources and reduced carbon sources were centrifuged to get the supernatants, and salting out method was used to precipitate proteins from the two supernatants, respectively. The precipitated proteins were dissolved in phosphate buffered saline (PBS), and the solutions were boiled for 5 min and then electrophoresed on sodium dodecyl sulphate polyacrylamide gel (SDS-PAGE). The electrophoresis was manipulated at a constant voltage of 120 V in separation gel (10%). The protein bands were stained with PAGE gel silver staining kit and identified on the basis of prestained protein ladder.

## Additional files

**Additional file 1: Table S1.** Physiological and biochemical characteristics of strain W12-1.

**Additional file 2: Table S2.** The nucleotide sequence of 16S rDNA from strain W12-1.

## Data Availability

The datasets used and/or analyzed during the current study are available from the corresponding author on reasonable request.
