# Supplementary material for: A newly isolated human intestinal bacterium strain capable of deglycosylating flavone C-glycosides and its functional properties
Source: Microb Cell Fact. 2019 May 28;18:94. doi: 10.1186/s12934-019-1144-7 (PMC6537369; doi:10.1186/s12934-019-1144-7)
Supplement: Supplementary file 1 — Additional file 1: Table S1. Physiological and biochemical characteristics of strain W12-1. [file 12934_2019_1144_MOESM1_ESM.doc]

Table S1. Physiological and biochemical characteristics of strain W12-1

| Utilization | Substrates |
| --- | --- |
| Positive reaction | Alanine-phenylalanine-proline aromase, D-amygdalin, methyl-β-D-glucoside, L-aspartate aromaminase, α-mannase, L-proline aromase, L-pyrrolidone aromase, tyrosine aromase, D-galactose, D-sorbitol, D-mannose, D-ribose, lactose, D-maltose, N-acetyl-D-glucosamine, D-mannitol, salicin, D-trehalose |
| Negative reaction | Phosphatidyl phospholipase C, arginine dihydrolase I, D-xylose, β-galactosidase, α-glucosidase, β-galactose glucosidase, phosphatase, leucine aromase, β-glucuronase, α-galactosidase, alanine aromaminase, urease, cyclodextrin, D-raffinose, amylopectin, sucrose, arginine dihydrolase II |
