# Supplementary material for: A newly isolated human intestinal bacterium strain capable of deglycosylating flavone C-glycosides and its functional properties
Source: Microb Cell Fact. 2019 May 28;18:94. doi: 10.1186/s12934-019-1144-7 (PMC6537369; doi:10.1186/s12934-019-1144-7)
Supplement: Supplementary file 2 — Additional file 2: Table S2. The nucleotide sequence of 16S rDNA from strain W12-1. [file 12934_2019_1144_MOESM2_ESM.doc]

Table S2. The nucleotide sequence of 16S rDNA from strain W12-1

tcccgagtgc ttgcactcaa ttggaaagag gagtggcgga cgggtgagta acacgtgggt 60

aacctaccca tcagaggggg ataacacttg gaaacaggtg ctaataccgc ataacagttt 120

atgccgcatg gcataagagt gaaaggcgct ttcgggtgtc gctgatggat ggacccgcgg 180

tgcattagct agttggtgag gtaacggctc accaaggcca cgatgcatag ccgacctgag 240

agggtgatcg gccacactgg gactgagaca cggcccagac tcctacggga ggcagcagta 300

gggaatcttc ggcaatggac gaaagtctga ccgagcaacg ccgcgtgagt gaagaaggtt 360

ttcggatcgt aaaactctgt tgttagagaa gaacaaggac gttagtaact gaacgtcccc 420

tgacggtatc taaccagaaa gccacggcta actacgtgcc agcagccgcg gtaatacgta 480

ggtggcaagc gttgtccgga tttattgggc gtaaagcgag cgcaggcggt ttcttaagtc 540

tgatgtgaaa gcccccggct caaccgggga gggtcattgg aaactgggag acttgagtgc 600

agaagaggag agtggaattc catgtgtagc ggtgaaatgc gtagatatat ggaggaacac 660

cagtggcgaa ggcggctctc tggtctgtaa ctgacgctga ggctcgaaag cgtggggagc 720

aaacaggatt agataccctg gtagtccacg ccgtaaacga tgagtgctaa gtgttggagg 780

gtttccgccc ttcagtgctg cagcaaacgc attaagcact ccgcctgggg agtacgaccg 840

caaggttgaa actcaaagga attgacgggg gcccgcacaa gcggtggagc atgtggttta 900

attcgaagca acgcgaagaa ccttaccagg tcttgacatc ctttgaccac tctagagata 960

gagctttccc ttcggggaca aagtgacagg tggtgcatgg ttgtcgtcag ctcgtgtcgt 1020

gagatgttgg gttaagtccc gcaacgagcg caacccttat tgttagttgc catcatttag 1080

ttgggcactc tagcgagact gccggtgaca aaccggagga aggtggggat gacgtcaaat 1140

catcatgccc cttatgacct gggctacaca cgtgctacaa tgggaagtac aacgagtcgc 1200

tagaccgcga ggtcatgcaa atctcttaaa gcttctctca gttcggattg caggctgcaa 1260

ctcgcctgca tgaagccgga atcgctagta atcgcggatc agcacgccgc ggtgaatacg 1320

ttcccgggcc ttgtacacac cgcccgtcac accacgagag tttgtaacac ccgaagtcgg 1380

tgaggtaacc tttttgga 1398
